# In Situ Optical Microspectroscopy Study of Isothermal Bleaching of γ-Irradiated International Simple Glass

**DOI:** 10.1021/acsphyschemau.3c00020

**Published:** 2023-07-26

**Authors:** Mariana Sendova, José A. Jiménez, Charles L. Crawford

**Affiliations:** † Optical Spectroscopy & Nano-Materials Lab, New College of Florida, Sarasota, Florida 34243, United States; ‡ Department of Chemistry & Biochemistry, Georgia Southern University, Statesboro, Georgia 30460, United States; § Savannah River National Laboratory (SRNL), Aiken, South Carolina 29808, United States

**Keywords:** activation energy, borosilicate glass, gamma radiation, *in situ* spectroscopy, thermal processing

## Abstract

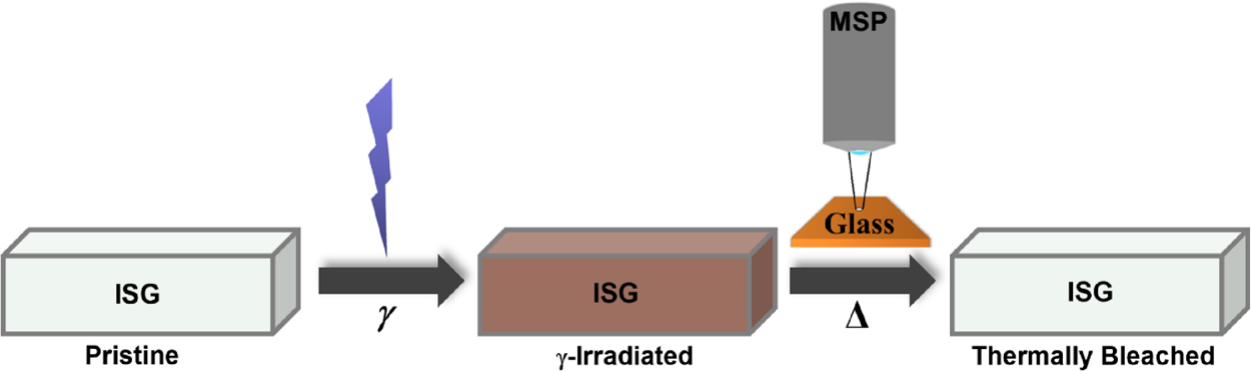

The isothermal bleaching of γ-irradiated glass was studied at elevated temperatures (280–340 °C) by real-time *in situ* optical microspectroscopy for the first time. The study was performed on γ-irradiated (0.83 and 1.99 MGy) International Simple Glass (ISG) borosilicate nuclear waste simulant made by Mo-SCI Corporation (Rolla, MO, USA). The current investigation proposes real-time optical transmission methodology for the activation energy assessment of isothermal bleaching of γ-irradiated glass. The method is based on robust quantification of the Urbach energy decay rates and yields similar activation energies for both doses within ∼0.24–0.26 eV.

## Introduction

The effects of ionizing radiation on various glass systems have been studied by various researchers for several decades.^1−12^ Apart from fundamental interest, applications in the areas of radiation dosimetry^4−10^ as well as evaluating the impact of γ-ray irradiation on glasses for nuclear waste vitrification have attracted much attention.^3,9,10,13−15^ The latter is of paramount importance within environmental management, and impacts generations to come as the glass forms are employed for immobilizing radionuclides for future disposal in a geological repository.^16^ Consequently, various groups have studied the influence of gamma radiation on various physical and chemical properties of borosilicate glasses aiming to understand the long-term behavior of the high-level radioactive waste.^3,9,10,13−15^

In this setting, the simplified borosilicate glass system with target composition (mass %) as 56.2SiO_2_-17.3B_2_O_3_-6.1Al_2_O_3_-12.2Na_2_O-5.0CaO-3.3ZrO_2_ known as International Simple Glass (ISG) was proposed as a simulant for doing comparisons throughout laboratories across different countries.^16,17^ The ISG originally produced by Mo-SCI Corporation (Rolla, MO, USA) was distributed and subsequently characterized by various means such as X-ray diffraction, thermal analysis techniques, chemical durability through the product consistency test, vibrational spectroscopy (e.g., Raman scattering), optical absorption, and photoluminescence (PL) spectroscopy.^17,18^ In ref (15), the authors reported on the influence of γ-ray irradiation on the original ISG available at the Savannah River National Lab (SRNL, Aiken, SC, USA). The glass was subjected to γ-ray irradiation with a Co-60 source up to an accumulated dose of ∼2 MGy, and the structural, thermal, corrosion, and optical properties were studied.^15^ The formation of the radicals ≡Si–O• (silicon-related non-bridging oxygen hole center, NBOHC) and/or ≡B–O• (boron-oxygen hole center, BOHC) by the γ-ray photons was then considered supported by electron paramagnetic resonance (EPR) spectroscopy, i.e., as
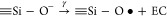
1
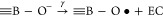
2where the electrons ejected from non-bridging oxygens are trapped in the glass forming an electron center (EC) defect. As the glass contained considerable iron as impurity, the formation of a (Fe^3+^)^−^ EC was also contemplated.^15^ Ultimately seeking to better understand the radiation-induced changes and further assess reversibility, an optical evaluation following an additional thermal treatment was also performed for the glass subjected to ∼2 MGy.^15^ The temperature was chosen near the *T*_g_ at 570 °C with a duration of 5 min, which resulted in the prompt color bleaching, the resulting glass sample thus resembling the pristine ISG. Hence, it was considered that if the γ-ray irradiation caused the NBOHC (or BOHC) and EC defects, their instability led to the electron–hole recombination processes triggered thermally (Δ) as
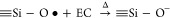
3
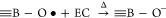
4

The thermal bleaching effect has been also reported previously by other researchers,^3,14^ indicating that glass annealing led to electron–hole recombination in γ-irradiated borosilicate glasses. Color bleaching was also observed for heat-treated phosphate glasses previously γ-irradiated.^11,19^ However, following the thermal bleaching effect in real time by optical spectroscopy has not been yet reported to the best of the authors’ knowledge.

*In situ* optical microspectroscopy has been proposed by the authors as a novel technique for studying metal nanoparticle precipitation in real time during thermal treatments at elevated temperatures.^20−23^ This has led to advances in understanding the relationship between thermal processing and resulting optical properties in connection to the underlying mechanisms governing the transformations of the embedded metallic phase (e.g., reduction of ionic species, kinetics of particle nucleation and growth). Hence, the technique was considered promising for obtaining new insights into the effects of thermal treatment unraveling the light–matter interaction involving the gamma photons affecting the nuclear waste simulant. Consequently, the current work was carried out employing the *in situ* optical microspectroscopy technique to study the thermal bleaching on the γ-irradiated ISG available at SRNL (Aiken, SC, USA). The experiments encompassed time-dependent isothermal studies, with data evaluated in the context of defects-related Urbach energies to gain insights into the energetics of the process.

## Experimental

The original ISG produced by Mo-SCI Corporation (Rolla, MO, USA)^17^ available at SRNL was used in this study, which was subjected to γ-ray irradiation to accumulated doses of 0.83 and 1.99 MGy as reported in ref (15). The doses are herein referred to for simplicity as ∼1 and ∼2 MGy. Details regarding gamma radiation experiments as well as the composition and properties of the pristine ISG and the γ-irradiated counterparts can be found elsewhere.^15,17,18^ The γ-irradiated ISG samples used in the present study were ∼1/16″ thick glass slabs that were polished for optical measurements.^15^

An analytical methodology was developed for assessing the activation energy of thermal bleaching of γ-irradiated glasses. Real-time optical transmission spectra were recorded at elevated temperatures (280–340 °C) with a CRAIC Technologies QDI 2010 microspectrophotometer (MSP) equipped with a Xe short-arc lamp and a Linkam THMS600 heating stage. The spectra were collected with a 10× objective, with square sampling aperture of 50 μm × 50 μm. Special attention was given to keeping the same sampling area during each experiment.

## Results and Discussion

**Figure 1:**
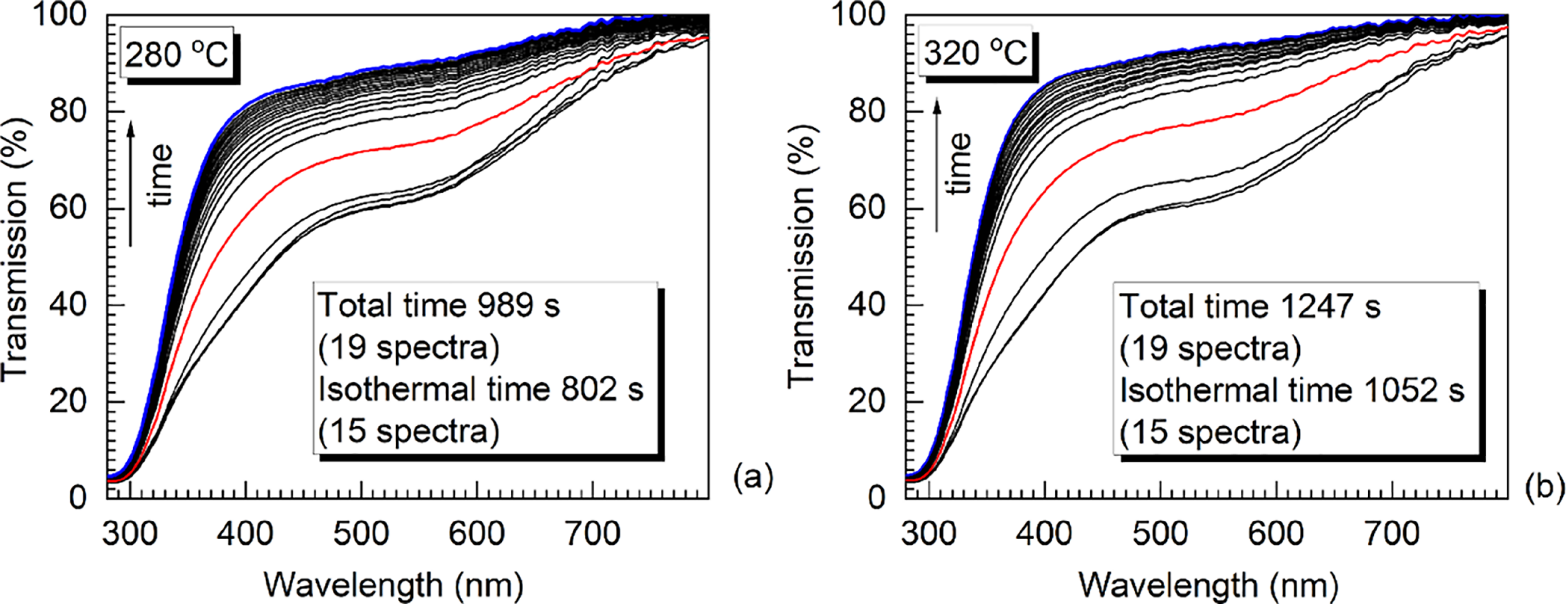
Transmission spectra time evolution of ∼2 MGy-irradiated ISG obtained under isothermal treatment at (a) 280 °C and (b) 320 °C. The isothermal heating onset is indicated with the red trace, and the end with a blue trace. The time and the spectra number taken during this time are indicated.

The spectral evolutions were followed for both γ-irradiation doses under isothermal treatments at 280, 300, 320, and 340 °C. As examples, shown in Figure 1a,b are the two sets of optical transmission spectra obtained isothermally at 280 and 320 °C, respectively, collected in real time *in situ* for ∼2 MGy-irradiated ISG samples. The temperature of the heating stage was initially ramped up at 100 °C/min to the target temperature, and then the glass sample was isothermally heated until the consecutive spectral changes became indiscernible. The acquisition time was ∼33 s. The spectra were collected every 38 (±2) s during the brief ramp up step (∼3 min) and increasing time intervals from 0.5 to 2 min during the isothermal step, up to ∼18 min. After 7 min, the spectral evolution slowed down significantly, and the rest of the spectra were collected at larger time intervals. The number of isothermally collected spectra for 280 and 320 °C are listed in Figure 1a,b, respectively. The transmission spectra during the ramp up are clustered together, while a significant jump is noticed for the final ramp up spectrum (red solid lines in Figure 1). The average temperature change during a single spectral acquisition in the ramp-up period is 62 (±2) °C. Visually, it is evident that during the thermal bleaching, the transmission spectra ‘bloom’ in a way that the trace opens more area with time as absorption decreases.

To further examine the process of thermal bleaching within the 280–340 °C range, the study proceeds with calculations and analysis of the Urbach energy, *E_U_*, for every transmission spectrum. *E_U_* is the characteristic parameter of choice since it allows understanding the microscopic physical processes in connection with the macroscopically measured spectra.^13−15,18,19^ In the glass being an insulator, strongly bound electrons reside in the valence band. This is a result of the localized electrons of the negatively charged non-bridging oxygens (NBOs) of the network former. The next highest band that the electrons can occupy is the conduction band, separated from the valence band by the so-called forbidden band gap. The energy difference between the localized and delocalized electron bands is the optical band gap.^24^ Whenever structural defects appear in the glass matrix, a possibility arises for the electrons to have energies, which are forbidden in an ideal glass matrix. Since glasses are structurally heterogeneous (amorphous), electron energies are not truly discrete but form an exponential tail of semicontinuous levels within the band gap. The parameter of Urbach energy is utilized for assessment of the width of these exponential tails as commonly carried out in the literature,^13,14^ as well as our previous works on the ISG.^15,18^ The analysis follows the relation of the absorption coefficient as
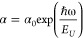
5where α_0_ is a constant and *ℏ*ω is the photon energy.^24,25^ The Urbach energy is then evaluated from the ln(α) *vs ℏ*ω plot, taking the reciprocal of the slopes of the linear portion of the plots.^13−15,18,19^ For consistency, the numerical procedure follows an algorithm for finding a tangential line of the steepest portion of the plot. The steepest section located *via* the maximum of the first derivative of the ln(α) versus *ℏ*ω plot. Further on, five points are utilized for a linear regression, Pearson’s factor ρ ∼0.999. On average, the ∼1MGy-irradiated ISG has Urbach energies of 550 (±10) meV, while the ∼2MGy-irradiated ISG has Urbach energies of 660 (±10) meV (20% higher value). At temperatures within 300 to 340 °C, the thermal bleaching was achieved for ∼20 min (e.g., Figure 1b, top traces).

**Figure 2:**
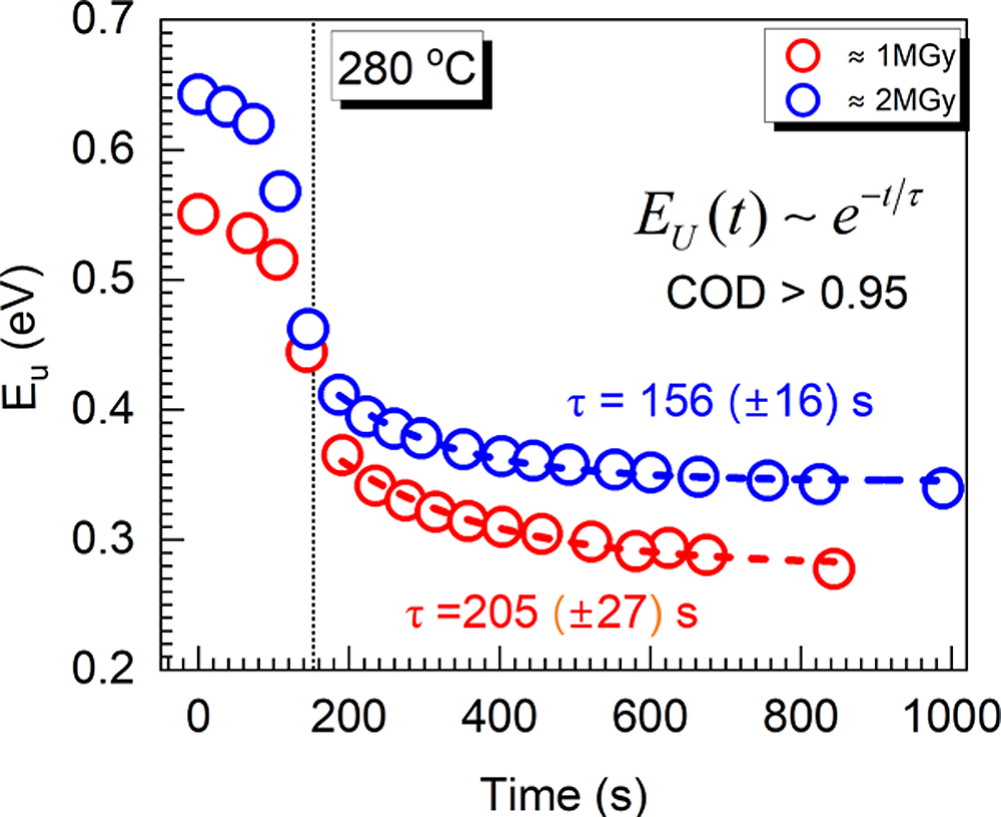
Urbach energy evolution for both doses of γ-irradiation with target temperature for isothermal bleaching at 280 °C. The onset of the 280 °C isothermal portion is indicated with vertical black dotted line; the fitting function (dashed lines) for the isothermal section, the COD, and the time decay parameters, τ, are listed.

The *E_U_* parameter can be implemented for the quantification of the defect states concentration and thus for the structural disorder of the material.^25^ Hence, we utilized the Urbach energy to follow the thermal bleaching process. The authors propose analyses of the real-time *in situ* time-dependent decay of the Urbach energy, *E_U_* (*t*), assessed for the two γ-irradiation doses at 280, 300, 320, and 340 °C. Plotted in Figure 2 are the *E_U_* values obtained following determinations *via*eq 5 for the isothermal treatments carried out at 280 °C for the two gamma radiation doses. The data suggests that ∼2/3 of the bleaching is completed during the brief temperature ramp-up period (the first ∼3 min, to the left of the reference line, Figure 2) and only ∼1/3 of the bleaching takes place during the isothermal heating, carried for an additional ∼18 min (to the right of the reference line, Figure 2).

The numerical analysis of the initial temperature ramp-up portion of the *E_U_* (*t*) data sets suggests that the inflection point of the Urbach energy evolution can be proposed as threshold bleaching temperature, at the used ramp rate of 100 °C/min. Therefore, the onset (threshold) bleaching temperature can be defined as the temperature at which the rate of bleaching is maximum (about −3 meV/s in the temperature interval up to 340 °C). Across all samples, the threshold temperature is 233 (±9) °C (44 meV) and does not seem to depend on the irradiation dose.

Numerical analysis of the *E_U_* (*t*) data reveals an exponential decay during the isothermal heating (dashed lines in Figure 2). The fitting equation is
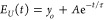
6The coefficient of determination (COD) of the exponential fit is >0.95 for all fits. The estimated decay times for all four temperatures are summarized in Table 1. It is noticed that the assessed isothermal decay times vary slightly with temperature, and it is of note that their confidence intervals do not overlap. In addition, the data listed in Table 1 suggests that the *E_U_* (*t*) decay rates of ∼2 MGy-irradiated ISG is consistently ∼75% faster for all temperatures.

**Table 1:** Decay Times in Seconds (s) Estimated for Different Isothermal Treatment Temperatures by the Exponential Fit, eq 6

	decay times at different temperatures			
γ-irradiation dose	280 °C	300 °C	320 °C	340 °C
∼1 MGy	205 (±27) s	170 (±19) s	155 (±20) s	122 (±13) s
∼2 MGy	156 (±16) s	130 (±20) s	112 (±16) s	90 (±14) s

**Figure 3:**
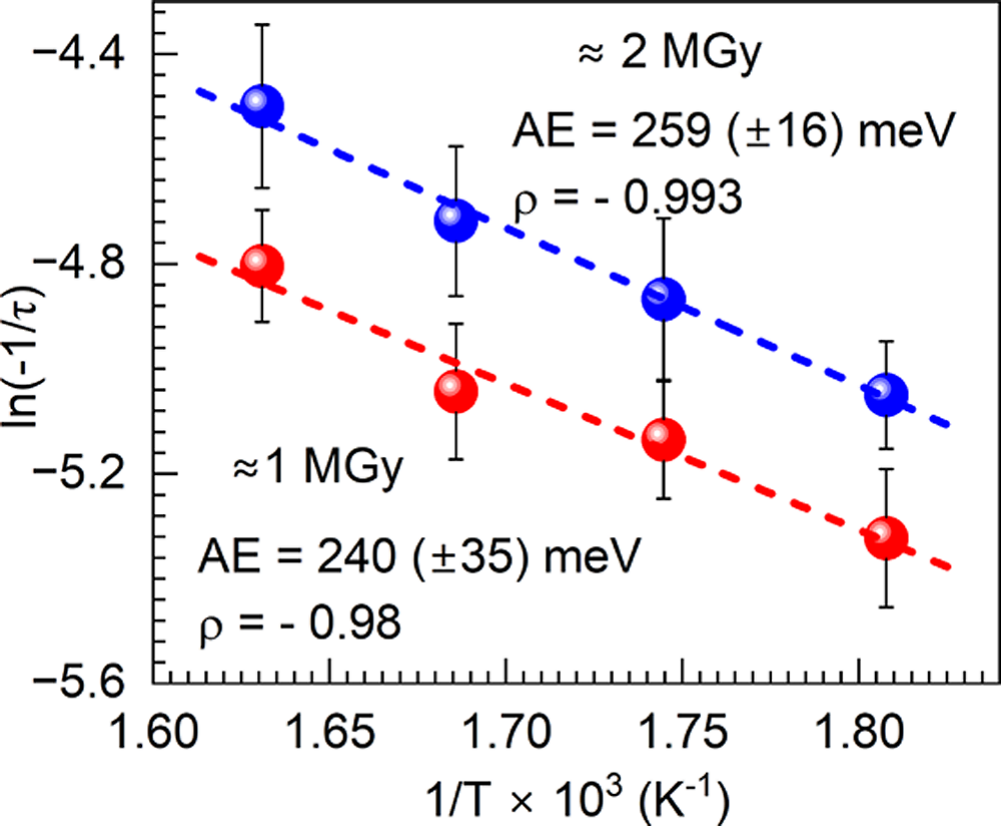
Arrhenius-type plots for the two doses of irradiation, utilizing the decay rates assessed with their uncertainties (vertical error bars); Pearson’s factor ρ and assessed activation energy (AE) values are listed.

Further on, the logarithm of the rates is plotted in an Arrhenius plot, i.e., *vs* 1/T (K^–1^), as shown in Figure 3. The Arrhenius consideration suggests that overall, the isothermal activation energy (AE) of bleaching appears to be similar for both doses, namely, at 240 (±35) and 259 (±16) meV for ∼1 MGy and ∼2 MGy, respectively. The uncertainties are derived from the regression slopes. The two values’ difference is less than the sum of their confidence intervals, deeming the isothermal activation energy independent of the γ-irradiation dose. The reported experimental values are in good agreement with the ab initio calculations of the migration of small polarons in olivine phosphate materials.^26^ The authors reported that their calculations yield similar, moderately low activation energy of 215 meV, suggesting high intrinsic polaron hopping rates.^26^

To interpret the activation energy values, the microscopic physicochemical processes are considered. During the irradiation, the flux of γ-photons ejects electrons from NBOs, which are consequently trapped by matrix defects or by the positively charged modifiers. Two types of defects’ energy levels are created within the band gap: (1) energy levels of the NBOHC defects – hole-trapping states; and (2) energy levels of trapped electrons. Since glasses are structurally heterogeneous, they support traps of depths from near zero to an upper limit of ∼1 eV.^26^ Overall, heating stimulates electron excitation from the electron-trapping states. Then, the freed electrons together with their self-induced local glass matrix bond distortions create local potential wells. The formed quasiparticles are called polarons. It is known that the polaron transport mechanism is hopping. Heat stimulates the polaron hopping rates. Thermally increased polaron hopping rates are detected in the proposed study (Table 1). Further on, in addition to the temperature dependence, the hopping rates are suggested^27,28^ to be influenced by the concentration of the defect sites in the glass matrix. At closer inspection of the data in Table 1, it is evident that longer *E_U_* decay times are associated with the glass with lower defect concentration (∼1 MGy). Ultimately, the polaron concentration vanishes due to recombination with the trapped holes in the NBOHC, recovering the negatively charged NBOs. Monte Carlo simulations suggest that different hopping processes for small polarons are influenced by sample composition and temperature.^27,28^ The decaying nature of the Urbach energy studied herein most probably is due to the interplay of varied hopping mechanisms and recombination rates of both defect types, yielding the detectable temperature-dependent lifetime of the γ-irradiation created electron polarons. The lifetime of the studied polarons is long (Table 1) in comparison to the photo-induced polarons studied by transient absorption spectroscopy.^27^ However, the reported herein polaronic activation energy average, ∼250 meV, is in good agreement with the experimental and theoretical ones reported in the literature.^26−29^

**Figure 4:**
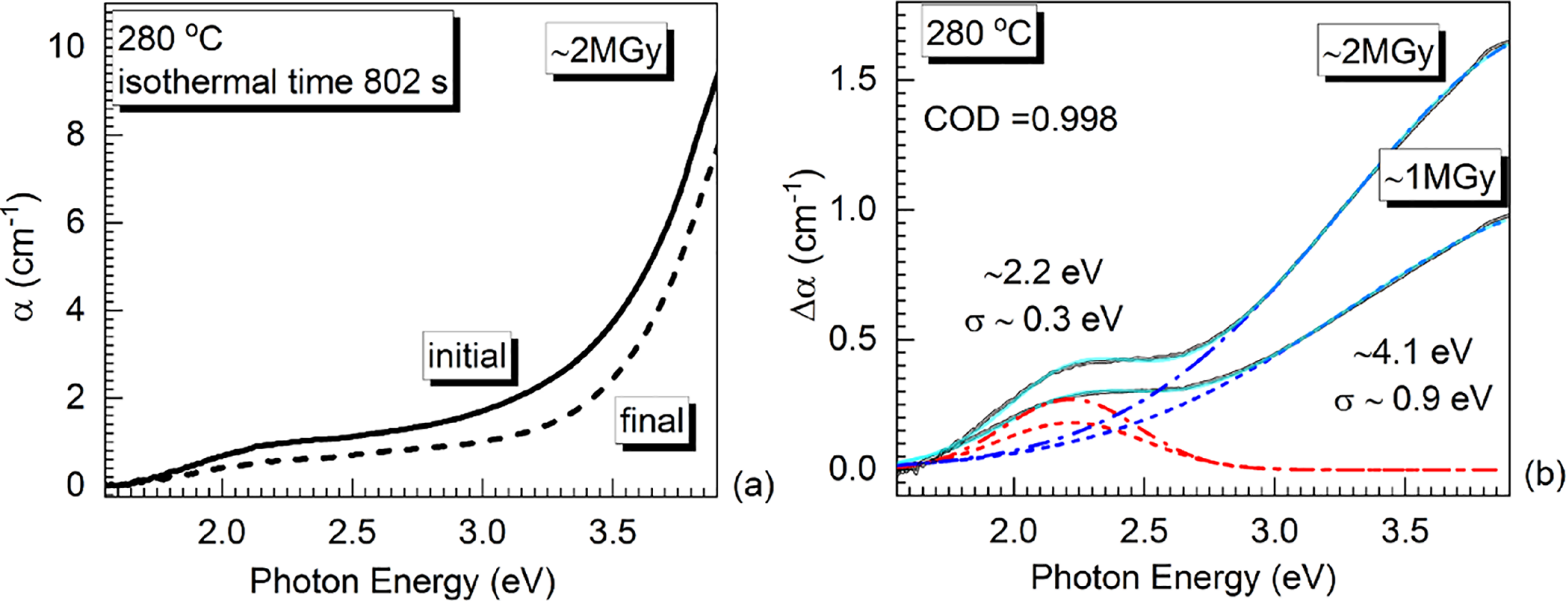
(a) Initial and final isothermal absorption spectra at 280 °C for the ∼2 MGy-irradiated ISG. (b) Difference absorption spectra, Δα (double black line); the irradiation dose is listed next to the traces. Panel (b) also shows a two-component Gaussian fit (red and blue dash lines for ∼1 MGy, red and blue dashed-dotted lines for ∼2 MGy, and cumulative spectral fits are cyan solid lines); the position, the standard deviation of the Gaussian fits, and the COD are listed.

To corroborate the AE energy discussion with the two trap-states model suggested by Paige,^30^ we expand the study with the goal of exploring the difference absorption spectra before and after the isothermal bleaching. Figure 4a illustrates the absorption spectra for the ∼2 MGy-irradiated ISG at 280 °C collected initially and after 802 s. The difference spectra at 280 °C for both doses are compared in Figure 4b (double black solid line). The authors propose that the difference spectra, Figure 4b, represent the thermally unstable (metastable, bleachable) spectral component. The ∼2 MGy-irradiated glass was monitored isothermally for 802 s and the ∼1 MGy-irradiated for 609 s. The fitting reveals two main Gaussian bands positioned at (1) ∼2.2 eV, with standard deviation, σ ∼0.3 eV, and (2) ∼4.1 eV, with σ ∼0.9 eV. The standard deviation of the Gaussian bands is proposed to be proportional to the depth of the electron and hole-trap energy levels. The lower energy absorption band at ∼2.2 eV seems to concur with the reported bleachable ‘A band’ in irradiated quartz, 2.3 eV.^30^ The broad, higher energy band at ∼4.1 eV is close to the Tauc assessment of the optical band gap of the pristine ISG at room temperature reported at 3.71 eV.^18^ The existence of a bleachable absorption band in close proximity to the optical band gap of pristine ISG suggests the γ-irradiation created shallow electron-trap levels around the conduction band edge (blurring the conduction edge). In addition, based on the bleachable intensity of the ∼4.1 eV absorption bands, Figure 4b, and the corresponding isothermal times (802 s for ∼2 MGy and 609 s for ∼1 MGy), the ∼4.1 eV band (∼2 MGy, ∼1 MGy) decay rate ratio was assessed to be temperature-independent (similar to the Urbach energy decay rates) with a value of ∼78%. The assessed ratio strongly agrees with the independently assessed *E_U_* (*t*) decay rate ratio, ∼75% discussed above (Table 1). Most importantly, the two-band deconvolution of the difference spectrum, Figure 4b, justifies the AE discussion in the framework of two trap-states model suggested previously by Paige.^30^

## Conclusions

In brief, this study proposed an isothermal real-time optical UV–Vis transmission methodology for activation energy assessment of γ-irradiated borosilicate glass thermal bleaching. The proposed technique followed the Urbach energy evolution with time (280–340 °C range) applied to the ISG produced by Mo-SCI Corporation (Rolla, MO, USA), which was irradiated at accumulated doses of 0.83 and 1.99 MGy. The Urbach energy decay rates were quantified and appeared to be consistently higher (∼75%) for the larger γ-irradiation dose in connection with the higher concentration of defects increasing the polaronic hopping rate. The technique yielded similar thermal bleaching activation energies values for both doses, namely, at 240 (±35) and 259 (±16) meV for 0.83 and 1.99 MGy, respectively. The deconvolution of the difference absorption spectra supported the two trap states model for γ-irradiated glasses. The decaying nature of the Urbach energy studied herein reflects the interplay between the defect- and temperature-modulated polaronic hopping mechanisms. The proposed method is applicable for the real-time monitoring and robust quantitative comparison of thermal bleaching of any γ-irradiated glassy material.
